# Distribution of white spot lesions among orthodontic patients attending teaching institutes in Khartoum

**DOI:** 10.1186/s12903-017-0380-7

**Published:** 2017-05-25

**Authors:** Maha Kamal Eltayeb, Yahia Eltayeb Ibrahim, Ikhlas Ali El Karim, Nada Mirghani Sanhouri

**Affiliations:** 1 Department of Oral Rehabilitation, Faculty of Dentistry, University of Khartoum, Khartoum, Sudan; 2 Dental School, Queen’s University Belfast, Northern Ireland, UK

**Keywords:** Fixed orthodontic appliances, ICDAS, Oral hygiene, Salivary calcium and phosphorus, White spot lesion

## Abstract

**Background:**

Fixed orthodontic appliances render teeth cleaning arduous, thus when orthodontic treatment is associated with inadequate oral hygiene practice, development of white spot lesions (WSLs) imposes a significant risk on the dentition. Salivary reservoir of calcium, magnesium, phosphorous and fluoride counteracts demineralization and encourages remineralization providing protection against caries challenge. The investigation of the factors leading to WSLs’ development is mandatory for appropriate prevention strategies planning. The present study aimed at evaluating the prevalence, pattern of distribution and contributing factors to WSLs’ development, among orthodontic patients attending orthodontic departments in teaching institutes in Khartoum.

**Methods:**

This cross-sectional descriptive, analytical clinical based study was carried out among fixed orthodontic patients attending teaching institutes in Khartoum State. All patients visiting the clinics for their follow up during a 3 months period and fulfilling the inclusion criteria were included. The International Caries Detection and Assessment System (ICDAS) served as a guide for standardized visual caries assessment. Saliva samples were collected from a sample of patients and the levels of calcium and phosphorus were measured. Patients were interviewed regarding their oral hygiene habits (frequency of tooth brushing, use of interdental brushes and mouth washes). Frequency distribution tables as well as graphs, Pearson’s correlations and Spearman’s correlation were used in the statistical analysis.

**Results:**

The overall prevalence of WSLs was 61.4%. The prevalence for each tooth was: 48.1% in the canine, 32.3% in the lateral incisor, 31.6% in both the central incisor and the first premolar, 27.2% in the second premolar and 8.9% in the first molar. No significant relationship between WSLs prevalence, age and gender or oral hygiene measures was found. There was no significant difference in calcium and phosphorus level between participants with WSLs or those with sound teeth (*p*-values for calcium and phosphorus were 0.154 and 0.567 respectively).

**Conclusions:**

Within the limitations of this study it was found that WSLs among fixed orthodontic patients represented an issue of concern. High prevalence of WSLs was recorded among orthodontic patients in Sudan, indicating a need for more stringent prevention programmes and oral hygiene practices prior to initiation of orthodontic treatment.

## Background

Enamel caries is typically first seen as an opaque, white color referred to as a White Spot Lesion (WSL) [1, 2]. WSLs are areas of subsurface demineralization beneath dental plaque [1]. Over time, the white spot may remineralize, but the opaque color usually remains, rendering the teeth unaesthetic [2].

The development of WSLs is attributed to prolonged plaque accumulation [3, 4]. Other factors aiding in development and progression of WSLs include: diet, deficiency in calcium, phosphate, fluoride and bicarbonate levels in saliva; medical and dental problems as well as genetic susceptibility [5, 6].

WSLs can either be carious or non-carious. For identification and diagnosis, teeth must be cleaned and dried prior to evaluation; using magnification and adequate light. Carious WSLs appear rough, opaque and porous. Non-carious lesions are mostly smooth and shiny [4]. Non-carious WSLs embrace fluorosis, developmental enamel hypomineralization and enamel hypoplasia.

Orthodontic treatment significantly amplifies the risk of WSLs when associated with inadequate oral hygiene [7]. Fixed orthodontic appliances create areas for food and plaque accumulation rendering tooth cleaning arduous leading to caries development [2, 3, 8–13]. Several reports have shown an increased incidence of WSLs on the labial enamel surface during orthodontic treatment [6–10] that begets a rapid increase in the volume of dental plaque with a lower pH, when compared to non-orthodontic patients [14, 15].

WSLs are clinically detected within a span of 4 weeks, which is typically the time period between one orthodontic appointment and the other [11, 16–18]. This should be enthralling for both patients and clinicians. Thus, to prevent tooth decay and unsightly tooth discolorations; prevention, early diagnosis and treatment of WSLs is crucial [18].

Depending on the examination techniques, the estimates of the prevalence of WSLs varied [3]. Estimates of the overall prevalence of WSLs arising during fixed orthodontic appliance therapy ranged widely from 2 to 96% [9, 11, 19, 20]. Studies where the teeth have been examined using visual scales have shown that more than 50% of subjects may experience an increase in the number of WSLs with fixed orthodontic appliances therapy [8–10].

The first line of defense against the development of WSLs has traditionally been patient education; emphasizing on optimal oral hygiene and proper diet. Fluoridated mouth rinses and varnishes, together with increased interest and development in calcium phosphate-based remineralization technology, have now been advocated as contemporary methods of prevention [21].

The International Caries Detection and Assessment System (ICDAS) is a clinical visual scoring system for use in dental education, clinical practice, research and epidemiology. It serves as a guide for standardized visual caries assessment [22, 23]. It defines six stages of the carious process, ranging from the early clinically visible changes in enamel to extensive tooth cavitation [24–27].

The aim of this study was to evaluate the prevalence and pattern of distribution of white spot lesions (WSLs) among orthodontic patients attending orthodontic departments in teaching institutes using the visual examination method “ICDAS” and aiming also at correlating the presence and pattern of WSLs with age of the patient, gender, duration of orthodontic treatment, frequency of visiting orthodontist and patients’ hygiene habits as well as correlating levels of calcium and phosphorus in patients’ saliva to the prevalence of WSLs.

## Methods

A cross sectional descriptive, analytical clinical based study was performed on patients starting fixed orthodontic treatment at the three orthodontic clinics of teaching institutes at Khartoum State. **Inclusion criteria**: Patients with fixed orthodontic appliances on labial tooth surfaces and patients who are on treatment for at least 1 month. **Exclusion criteria**: Patients with fixed orthodontic appliance on lingual surfaces, patients with fixed orthodontic appliances for less than 1 month, and patients with rampant caries. The study was approved by the University of Khartoum, Faculty of Dentistry Research Ethics Committee (Meeting No. 3; Date 16/9/2014).

### Clinical procedures

All patients who visited the clinics for their follow up during the 3 months period of the study (September 2014 to November 2014) and fulfilled the inclusion criteria were invited to participate in the study and an informed written consent was obtained from all the participants. All participants were interviewed for personal and socio-demographic variables including name, age and gender and general health status.

The clinical examination was performed by two experienced dentists who received “ICDAS” e-learning on how to use this system and were also calibrated in performing the clinical assessment.

Examination was carried out on patients who were on fixed orthodontic treatment for at least 1 month. Teeth were cleaned before examination using prophylaxis paste and brush, then, visual examination was carried out under standard light conditions on the dental chair. The facial surfaces of maxillary and mandibular teeth from the right first molar to the left first molar were examined. Teeth were isolated with cotton rolls and air-dried for 5 s prior to examination. Only teeth surfaces gingival to the arch-wire were examined for the presence of WSLs, as this is the area most prone to enamel demineralization during orthodontic treatment [3]. Lesions were coded according to visual examination criteria “ICDAS” from zero to two. The following scale was used for the visual examination (Table 1).

**Table 1:** Visual examination criteria “ICDAS”

0	Sound tooth surface: no evidence of caries after prolonged air drying (5 s)
1	First visual change in enamel: opacity or discoloration (white or brown) is visible after prolonged air drying
2	Distinct visual change in enamel: opacity or discoloration distinctly when wet, lesion must still be visible when dry

The total cases of fixed orthodontic appliance during 3 months were 220. All patients who visited the clinic for their follow up during the 3 months period of the study and fulfilled the inclusion criteria were included.

The saliva samples were taken using the Slovin’s formula:

*n = N*/*(1 + Ne* ^*2*^ *) (The sample equation used when the population is known; the total population was 220)*

Where:

n is the sample size

N is population size

e is the margin of error which is 0.1 (10%)

So applying the above formula the sample size will be:

$$ \boldsymbol{n} = \frac{220}{1+{\mathrm{220.0.1}}^2}=69\ \mathrm{patients} $$

Systematic random sampling technique was used.

### Salivary collection

Following clinical assessment salivary samples were collected from the systematically randomly selected subjects. Participants were instructed to rinse their mouths thoroughly with water to remove any possible contaminating substances. Fifteen ml of paraffin wax stimulated saliva were then collected from the subjects into calibrated plastic containers and stored at 4 °C for laboratory analysis.

After salivary collection was completed and at the end of the interview, the patients with WSLs were given strict oral hygiene instructions and a fluoridated mouth wash was prescribed.

### Laboratory procedure

Salivary samples were analyzed for calcium and phosphorus levels. One ml of saliva was diluted in 10 ml of nitric acid 1% [28], and then the calcium level was measured using atomic absorption spectrophotometery (atomic absorption spectrophotometer model 210 VGP, Black scientific, Norwalk city, USA) as previously described. Similarly phosphate levels were measured using spectrophotometery (Spectronic 20, Milton Roy company, Warminster city, England) as described by Tandon [29]. The technique involved addition of 4 ml of saliva to 10 ml of reagent (the reagent prepared by mixing ammonium molybdate and potassium antimony tartrate and ascorbic acid with specific weights) and the volume was increased to 50 ml using distilled water.

### Statistical analysis

Data was analyzed using descriptive statistics including frequency distribution tables as well as graphs. Pearson’s correlation was used to correlate WSLs and selected socio-demographic variables and other health variables whereas Spearman’s correlation was used to correlate the levels of calcium and phosphorus with the presence of WSLs. For all tests a *p*-value <0.05 was considered significant. Kappa test was done for intra- and inter-examiner agreement and reliability.

## Results

A total of 158 patients were examined making a response rate of 71.8%.

For inter and intra examiner agreement kappa test was used and the *p*-value was < 0.01 indicating excellent agreement.

The overall prevalence of WSL was 61.4%. The prevalence in each tooth was: 48.1% in the canine, 32.3% in the lateral incisor, 31.6% in both the central and the first premolar, 27.2% in the second premolar and 8.9% in the first molar (Table 2).

**Table 2:** Prevalence of WSL in each tooth type (*n* = 158; 948 teeth)

		No.	Percent
First molar	Sound tooth	144	91.1%
	First or second visual change in enamel	14	8.9%
Second premolar	Sound tooth	115	72.8%
	First or second visual change in enamel	43	27.2%
First premolar	Sound tooth	108	68.4%
	First or second visual change in enamel	50	31.6%
Canine	Sound tooth	82	51.9%
	First or second visual change in enamel	76	48.1%
Lateral incisor	Sound tooth	107	67.7%
	First or second visual change in enamel	51	32.3%
Central incisor	Sound tooth	108	68.4%
	First or second visual change in enamel	50	31.6%

Age was divided into groups and the group (>30 year) have shown the highest percentage of WSLs (Table 3). Pearson correlation coefficient was calculated for measuring the relationship between WSLs and age; no significant correlation between age and WSLs (*p*-value = 0.788) was found.

**Table 3:** Caries by age of the participating patients (*n* = 158)

Age group		Caries		Total
		No	Yes	
11–20	Count	23	34	57
	%	40.4%	59.6%	100.0%
21–30	Count	35	54	89
	%	39.3%	60.7%	100.0%
>30	Count	3	9	12
	%	25.0%	75.0%	100.0%
Total	Count	61	97	158
	%	38.6%	61.4%	100.0%

The percentage of the female participants was 75.9% and that of the male participants was 24.1%. For the effect of gender on WSLs occurrence, Mann–Whitney non-parametric test was used. It showed no significant difference in WSLs index between the different genders (*p*-value 0.393). The percentage of females and males who developed WSLs was 46.6 and 14.8% respectively.

The percentage of the participants who go to the orthodontic clinic for their follow up visits every month was 99% and those who go every 3 months was 1%. There was not a correlation between frequency of follow up visits to the orthodontic clinic and WSLs (*p*- value 0.240).

The mean time for the participants in orthodontic treatment was 19.52 months with a minimum of 1 month and a maximum of 96 months. Most of the participants were on treatment for less than 2 years. There was no significant relation between WSLs and duration of orthodontic treatment (*p*-value 0.349).

All of the participants (100%) were using tooth brush and paste as a routine oral hygiene measure; 56% of them brush their teeth twice per day, 15% once per day, 22% three times a day, 6% four times a day and 3% 5times a day. About 63% were using interdental brush and 37% weren’t. Twelve percent of participants were using mouth wash and 88% weren’t. Frequency of brushing had no significant relation with presence of WSLs (*p*-value .195). Use of interdental brush and mouth washes also showed no significance. Mann–Whitney test was used and the *p*-value was 0.703 and 0.138 respectively.

The mean salivary calcium for participants who had no WSLs was 1.569 mg/l and for those who had WSLs was 1.378 mg/l. For phosphorus, the level for participants with WSLs was 6.821 mg/l, and for those with sound teeth was 6.329 mg/l. There was no significant difference in calcium and phosphorus level between participants with WSLs and those without (*p*-value for calcium was 0.154 and for phosphorus was 0.567). The number of cases with sound teeth was 17 and 38 is the number of cases of teeth with WSLs.

## Discussion

Based on clinical visual examination this study showed a high prevalence of WSLs (61.4%). Its’ results showed that WSLs remains a considerable problem during orthodontic treatment. Caries diagnosis and detection were carried out using ICDAS criteria. This criteria is very good in detecting early enamel changes [30]. The examination was carried out on clean dry teeth giving the early carious lesion two codes according to severity. In other studies a high prevalence of WSLs among orthodontic patients was also determined; for instance Mizrahi found a significant increase in prevalence of WSLs (72.3–84%) among fixed orthodontic patients [20] and Boersma et al. recorded a prevalence of 97% [31], using quantitative light induced fluorescence. Gorelick et al. reported a prevalence of 50% [9]. The sample size in the previous studies [10, 30] was less than in the current study. Gorelick et al. also used the photographs method which may be considered less accurate than direct clinical examination used in this study. It should be noted here that the photographs used by Gorelick were not digital, he used kodachrome slides but the use of digital radiography might have provided a good opportunity to improve outcomes as it allows for magnification and adjustment on the quality of the image, however the evidence from the literature suggests that the digital radiography and visual inspection are comparable in caries detection [2, 32].

The study showed that there was no significant difference between males and females regarding the presence of WSLs. This result concurred with many previous studies [20, 33–35]. The percentage of females who developed WSLs during treatment was higher (46.6) than the percentage of males (14.8), but the difference was statistically non significant. Regarding age of the patient, there was no significant association between it and the prevalence of WSL (*p*-value = .788). Most of the patients were aged between 21 and 30 years and 60.7% of them developed WSLs.

Canines were found to be the most affected teeth with WSLs, followed by lateral incisors, then the central incisors and first premolars with the same percentage, followed by the second premolars and lastly the first molars. Literature showed conflicting results on which tooth is more affected; Gorelick et al. indicated the maxillary lateral incisor had higher incidence for WSLs [9], while Geiger et al. showed that the maxillary lateral incisor and canines were the most commonly affected teeth [35]. On the other hand, Mizrahi concluded that the maxillary and mandibular first molars were the teeth most commonly affected. He examined two groups of patients, one group was pre-orthodontic treatment and the other was post-orthodontic treatment. He used visual examination and divided the teeth surface into thirds; incisal, middle and cervical thirds. The severity was scored from 0 to 3 [10]. In the current study the low prevalence of WSLs on the first molars may be due to difficulty of examination due to presence of the orthodontic bands.

Ninety-nine percent of participants reported visiting orthodontist for follow up on monthly basis. There was no statistical significant relation between frequency of visiting orthodontic clinic and WSLs.

The relationship between the duration period of orthodontic appliance and the prevalence of WSLs was statistically non significant (*p*-value = 0.349). In the present study the time was divided into 2 years groups and the highest percentage of WSLs was on 4–6years duration group. Julian et al. divided the duration into 24–36 months and >36 months, he found a significant difference in number of WSLs [2], while Lovrov et al. found no correlation between treatment time and WSLs [33].

The oral hygiene had no significant effect in the development of WSLs. It was evaluated depending on patient’s words; they were asked about their oral hygiene measures (if they use tooth brush and paste, interdental brush and mouth wash) and frequency of cleaning. There was however no specific question on whether participants have used fluoridated or non-fluoridated tooth paste limiting the information that could have resulted in showing some significance for the findings.

In a study that evaluated the effect of use of fluoridated toothpaste on the remineralization of WSLs after debonding there was significant difference between use of fluoridated and non fluoridated toothpastes in decreasing WSLs [1]. Other studies concluded that the regular use of fluoride toothpaste is a very common recommendation by the orthodontist, but it was shown to be inefficient in inhibiting white spot development around the orthodontic brackets [2, 3].

The results of this study may be considered in the light of its limitations. These limitations can be summarized as follows: Examination of the participants was carried out visually and this may be less sensitive than other techniques like light fluorescence. Oral hygiene of the patients was not measured clinically and information about oral hygiene measures was verbally collected from patients. The presence of the fixed orthodontic appliance during examination had increased the difficulty to visualize the lesions.

## Conclusions

Within the limitations of this study the followings could be concluded; this study was the first of its type to be done among Sudanese orthodontic patients. It recorded that WSLs among fixed orthodontic patients represented an issue of concern. The prevalence of WSLs was (61.4%). The most affected teeth were canines and lateral incisors. Although no significant relation between WSLs and age of the patient, gender, duration of orthodontic treatment, frequency of visiting orthodontic clinic and levels of calcium and phosphorus was found, patients undergoing fixed orthodontic treatment should be warned against the high risk of developing WSLs and eventual frank carious lesions, if proper oral hygiene measures were not ensued.

## Abbreviations

ICDAS: International caries detection and analysis system
WSL: White spot lesion
